# The Welsh study of mothers and babies: protocol for a population-based cohort study to investigate the clinical significance of defined ultrasound findings of uncertain significance

**DOI:** 10.1186/1471-2393-14-164

**Published:** 2014-05-08

**Authors:** Lisa Hurt, Melissa Wright, Fiona Brook, Susan Thomas, Frank Dunstan, David Fone, Gareth John, Sue Morris, David Tucker, Marilyn Ann Wills, Lyn Chitty, Colin Davies, Shantini Paranjothy

**Affiliations:** 1 Institute of Primary Care and Public Health, School of Medicine, Cardiff University, Neuadd Meirionnydd, Heath Park, Cardiff CF14 4YS, UK; 2 Aneurin Bevan University Health Board, Lodge Road, Caerleon, Newport NP18 3XQ, UK; 3 Public Health Wales NHS Trust, 14 Cathedral Road, Cardiff CF11 9LJ, UK; 4 NHS Wales Informatics Service, 12th Floor, Brunel House, 2 Fitzalan Road, Cardiff CF24 0HA, UK; 5 Cardiff and Vale University Health Board, Cardigan House, University Hospital of Wales, Heath Park, Cardiff CF14 4XW, UK; 6 National Childbirth Trust, Alexandra House, Oldham Terrace, London W3 6NH, UK; 7 UCL Institute of Child Health, London WC1N 1EH and Great Ormond Street and UCLH NHS Foundation Trusts London, 30 Guilford St, London, UK; 8 Cwm Taf University Health Board, Ynysmeurig House, Navigation Park, Abercynon, Rhondda Cynon Taff CF45 4SN, UK

**Keywords:** Ultrasound, Anomaly, Markers, Echogenic bowel, Cerebral ventriculomegaly, Renal pelvicalyceal dilatation, Nuchal thickening, Cardiac echogenic foci, Choroid plexus cysts, Short femur, Congenital abnormality, Stillbirths, Pre-term birth, Small for gestational age

## Abstract

**Background:**

Improvement in ultrasound imaging has led to the identification of subtle non-structural markers during the 18 – 20 week fetal anomaly scan, such as echogenic bowel, mild cerebral ventriculomegaly, renal pelvicalyceal dilatation, and nuchal thickening. These markers are estimated to occur in between 0.6% and 4.3% of pregnancies. Their clinical significance, for pregnancy outcomes or childhood morbidity, is largely unknown. The aim of this study is to estimate the prevalence of seven markers in the general obstetric population and establish a cohort of children for longer terms follow-up to assess the clinical significance of these markers.

**Methods/Design:**

All women receiving antenatal care within six of seven Welsh Health Boards who had an 18 to 20 week ultrasound scan in Welsh NHS Trusts between July 2008 and March 2011 were eligible for inclusion. Data were collected on seven markers (echogenic bowel, cerebral ventriculomegaly, renal pelvicalyceal dilatation, nuchal thickening, cardiac echogenic foci, choroid plexus cysts, and short femur) at the time of 18 – 20 week fetal anomaly scan. Ultrasound records were linked to routinely collected data on pregnancy outcomes (work completed during 2012 and 2013). Images were stored and reviewed by an expert panel.

The prevalence of each marker (reported and validated) will be estimated. A projected sample size of 23,000 will allow the prevalence of each marker to be estimated with the following precision: a marker with 0.50% prevalence to within 0.10%; a marker with 1.00% prevalence to within 0.13%; and a marker with 4.50% prevalence to within 0.27%. The relative risk of major congenital abnormalities, stillbirths, pre-term birth and small for gestational age, given the presence of a validated marker, will be reported.

**Discussion:**

This is a large, prospective study designed to estimate the prevalence of markers in a population-based cohort of pregnant women and to investigate associations with adverse pregnancy outcomes. The study will also establish a cohort of children that can be followed-up to explore associations between specific markers and longer-term health and social outcomes.

## Background

All pregnant women in the UK are offered an ultrasound scan to screen for structural anomalies between 18 and 20 weeks of pregnancy as part of their routine antenatal care [1]. Some structural abnormalities detected at this examination may be associated with an increased risk of chromosomal abnormalities. The diagnostic test for confirming this requires analysis of fetal genetic material that may be obtained by invasive procedures such as chorionic villi sampling or an amniocentesis which carry a procedure-related miscarriage risk of at least 1% [2].

In addition to significant structural abnormalities, ultrasound imaging has led to the identification of more subtle markers such as echogenic bowel, mild cerebral ventriculomegaly, renal pelvicalyceal dilatation, nuchal thickening, cardiac echogenic foci (golf balls), choroid plexus cysts, and short femur. In this protocol paper we refer to these collectively as markers. Reports of the prevalence of markers detected on antenatal scans vary, with one review suggesting that the range of the overall prevalence of all markers was 0.6% to 4.3% [3]. Isolated cardiac echogenic foci have been reported in 0.5%-4.9% of scans [4,5], choroid plexus cysts in 0.6% - 2.1% [6], mild pelvicalyceal dilatation in 0.3% - 4.5% [7,8] and echogenic bowel in 0.2% – 1.4% [7,9]. Some of this variation was attributed to heterogeneity in the populations that have been studied. Many studies were carried out in tertiary or specialist institutions that manage a large number of high-risk pregnancies, while in other studies there was a lack of information about the characteristics of the populations that were studied. Variation may also result from the use of different definitions of the markers.

The clinical significance of these markers is unclear [3]. For example, there have been reports that echogenic bowel may be associated with cystic fibrosis [9,10] and adverse pregnancy outcomes such as intra-uterine growth restriction and stillbirths [11,12], particularly in high-risk pregnancies. However, there is uncertainty about their significance in low-risk pregnancies (i.e. those who do not have risk factors for chromosomal abnormalities or other pregnancy complications). There is also uncertainty among healthcare professionals about the best practice in clinical management of pregnancies where these markers are identified at the fetal anomaly scan [13]. UK-wide data has suggested that there is variation between hospitals in their clinical management of markers and in the information and quality of care given to women [14,15].

The reporting of ultrasound findings of uncertain significance can cause unresolved maternal anxiety [16] and exposure to the risks associated with invasive diagnostic tests, although new standards for the performance of amniocentesis and chorionic villus sampling in Wales give clear guidance on appropriate indications for these procedures [17]. For some of these markers, it is not known whether there are long-term adverse health outcomes in children [3]. Questions that remain unanswered include: whether cardiac echogenic foci are associated with future development of cardiac abnormalities in childhood; whether mild cerebral ventriculomegaly (defined as a ventricular atrial diameter at any gestation of 10 mm to 12 mm) is associated with neuro-developmental delay and poorer educational outcomes; or whether pelvicalyceal dilatation is associated with postnatal uropathies or recurrent urinary tract infections in childhood.

We designed a population-based study to investigate the prevalence of markers and their associations with adverse pregnancy outcomes and longer-term health and social outcomes in children. The study objectives are:

1. To estimate the prevalence of individual markers at the 18 to 20 week anomaly scan in an unselected population of pregnant women attending for routine antenatal care in Wales;

2. To assess inter- and intra-sonographer variability in the detection of markers;

3. To investigate associations between the presence of markers and adverse pregnancy outcomes (major congenital abnormality, stillbirth, pre-term birth and small for gestational age);

4. To establish a cohort of children that can be followed-up to investigate associations between specific markers and longer-term health outcomes.

## Methods/Design

### Research design and setting

This is a population-based cohort study embedded within the routine antenatal services offered to pregnant women by NHS Wales. The population of Wales is approximately 3 million people, of whom approximately 20% are women of reproductive age [18]. All pregnant women have access to NHS maternity care, with almost universal uptake. Minimum standards for maternity care (including guidance on performing ultrasound scans) are recommended by the National Institute for Health and Care Excellence (NICE) and include at least ten antenatal check-ups for women expecting their first baby [1]. Wales is in a unique position to carry out this study because of the way in which healthcare data are routinely collected and can be electronically record-linked. First, an electronic information system for radiological data storage and reporting (Radiology Information Service 2 or RadIS2) was recently implemented in Welsh hospitals. Second, there are ongoing high-quality population-based registers for child health, including measures of pregnancy outcome such as gestation and weight at birth, congenital anomalies, stillbirths, neonatal and infant deaths recorded for all babies born in Wales. All of these systems (including RadIS2) use NHS numbers which can be used to link mother and baby across these routinely collected child health datasets. This provided a unique opportunity to collect standardised information on markers in a cohort of pregnant women and link these data to pregnancy and child health outcomes in their babies.

### Ethical approval

Ethical approval was given by the Multicentre Research Ethics Committee (MREC) for Wales (reference 08/MRE09/17).

### Participants and recruitment

#### *Inclusion and exclusion criteria*

All pregnant women receiving antenatal care within six of seven Welsh Health Boards who had an 18 to 20 week ultrasound scan in Welsh NHS Trusts between July 2008 and March 2011 were eligible for inclusion. The seventh Board was excluded as some of their population received antenatal ultrasound scans from hospitals outside Wales that did not use the RadIS2 system. Women were excluded if they were unable to give written informed consent.

#### *Sample size calculations*

Given the number of births in each Health Board and the planned phased introduction schedule for RadIS2 (which varied across Health Boards), it was anticipated that approximately 39,000 pregnant women would be eligible for recruitment during the study period. Assuming that 75% of these women would agree to participate (29,000) and that complete ultrasound and outcome data would be available for 80% of pregnancies, the study would have a projected sample size of 23,000.

A broad range of estimates for the prevalence of markers has been reported in previous studies (Table 1). Using several values from within this range, we performed sample size calculations to estimate i) the precision with which we could measure marker prevalence, and ii) the risk ratios that could be detected when examining the association between a marker and an adverse pregnancy outcome with 80% power at a 5% significance level. Data from 23,000 pregnancies would allow the prevalence of individual markers to be estimated with the following precision: a marker with 0.50% prevalence to within 0.10%; a marker with 1.00% prevalence to within 0.13%; and a marker with 4.50% prevalence to within 0.27%. The following sample size calculations are based on 80% power and a 5% Type 1 error rate. With 23,000 pregnancies and a marker prevalence of 0.5%, the sample size would be adequate to detect a 7-fold increase in an adverse pregnancy outcome which had a baseline prevalence of 1%, or a 2.75-fold increase in an adverse pregnancy outcome with a prevalence of 5%. With a marker prevalence of 1%, the sample size would be adequate to detect a 5-fold increase in an adverse pregnancy outcome which had a prevalence of 1%, or a 2-fold increase in an adverse pregnancy outcome with a prevalence of 5%. With a marker prevalence of 4.50%, the sample size would be adequate to detect with a 2.3-fold increase in an adverse pregnancy outcome which had a prevalence of 1%, or a 1.5-fold increase in an adverse pregnancy outcome with a prevalence of 5%.

**Table 1:** Definition of markers included in the study

			
1 Echogenic bowel (EB)		Areas of increased echogenicity in the fetal bowel that are as bright as bone. Single or multiple loops of bowel may be identified and it may be noted to be solid intraluminal echogenicity or occasionally echogenicity of the walls only (tram line).	Reported prevalence at fetal anomaly scan from previous studies [7,9]: 0.2 – 1.4%
2 Mild to moderate ventriculomegaly (VM)		Mild to moderate ventriculomegaly is a ventricular atrial diameter, at any gestation, from 10 mm to 15 mm. Measurements are obtained from a transventricular axial view at the level of the glomus of the choroid plexus. The callipers were placed on the inner margins of the echogenic ventricular wall.	Reported prevalence at fetal anomaly scan from previous studies [19]: 0.1%
3 Pelvicalyceal dilatation (PCD)		Fluid filled dilatation of the renal pelvis measured on axial section with an anterior-posterior (AP) diameter of 5 mm or greater (callipers to be placed on the inner AP margins of the pelvic wall). This may be unilateral or bilateral.	Reported prevalence at fetal anomaly scan from previous studies [7,8]: 0.3 – 4.5%
4 Nuchal thickening (NT)		Thickening of the skin and the subcutaneous tissues on the posterior aspect of the fetal neck. This is best viewed in a modified biparietal diameter (BPD) view to include the cavum septum pellucidum and cerebellum. Assessed by measuring the distance between the skin and occipital bone at the posterior aspect of the neck with the callipers placed on the outer edge of the bone and the outer edge of the skin. A measurement of 6 mm or greater was considered to indicate thickening before 20 + 6 weeks gestation.	Reported prevalence at fetal anomaly scan from previous studies [20,21]: 0.4 – 0.6%
5 Choroid plexus cysts (CPC)		Small sonographically discrete fluid-filled spaces ≥ 5 mm within the choroid plexus and seen on scan as black echo-free areas. May be single, multiple, unilateral or bilateral.	Reported prevalence at fetal anomaly scan from previous studies [6]: 0.6 – 2.1%
6 Echogenic cardiac foci (ECF)		Echogenic area on the papillary muscle of either (usually left) or both of the atrioventricular valves.	Reported prevalence at fetal anomaly scan from previous studies [4,5]: 0.5 – 4.9%
7 Short femur (SF)		Femur length which is below two standard deviations (3rd centile) for gestational age when measured with the shaft of the femur parallel to the transducer. Care must be taken to ensure that the entire diaphysis of the femur is measured. If the epiphyseal cartilages are visible they were not included in the measurement. It is assumed that the remainder of the skeleton is normal.	Reported prevalence at fetal anomaly scan from previous studies [7]: < 5%

#### *Recruitment*

**Figure 1:**
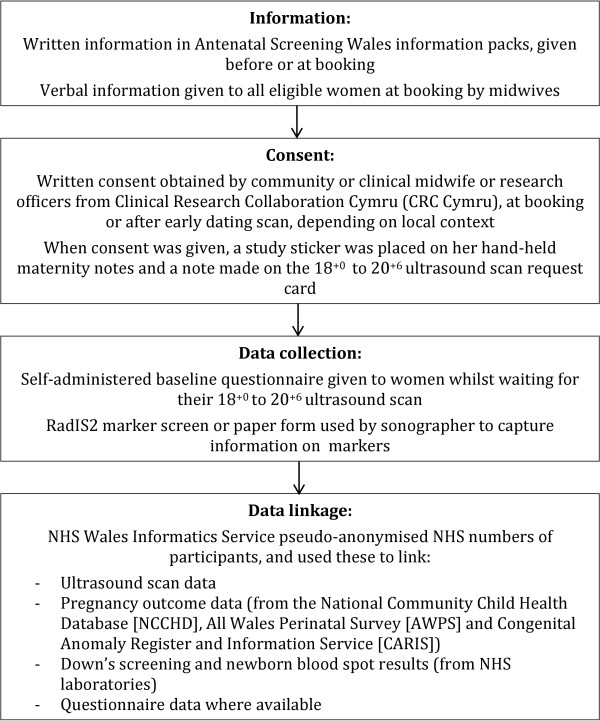
Flow chart of recruitment, data collection and data linkage.

Figure 1 shows the flow chart for recruitment, data collection and data linkage. Recruitment of pregnant women to the study started in July 2008 and ended in March 2011, with a phased roll-out to match the planned phased roll out of RadIS2 across Wales. A study information leaflet was included in the antenatal screening information pack given to pregnant women as early as possible in the pregnancy (at approximately eight to ten weeks of gestation). Women were given verbal information about the study and the opportunity to ask questions and obtain clarification about participation during their first antenatal visit with a healthcare professional (usually conducted by a community midwife between eight and twelve weeks of pregnancy). At this appointment, consent was obtained to:

1. Record the presence of markers;

2. Obtain antenatal Down’s syndrome screening results and cytogenetic results (if undertaken);

3. Use NHS numbers of the mother and her baby to link ultrasound data with data on pregnancy and health outcomes from the National Community Child Health Database (NCCHD), All Wales Perinatal Survey (AWPS), Congenital Anomaly Register and Information Service (CARIS), and newborn bloodspot tests; and,

4. Make future contact to obtain follow-up data on their baby.

Study stickers were placed on the front of the antenatal maternity hand-held notes of all women who had consented to participate to aid in the identification of these women at future contacts with health professionals. Stickers were also placed on the request card for the 18 to 20 week ultrasound scan in some sites. The consent form and the patient information leaflet are shown in Additional file 1 and Additional file 2.

#### *Baseline data collection*

The specific data items collected and the sources of data are shown in Table 2. A self-administered questionnaire following recruitment was used to collect baseline data on socio-demographics, smoking and alcohol use, and obstetric history. The postcode was used to assign women into a deprivation quintile using the Welsh Index of Multiple Deprivation [22]. In all Health Boards except one, women were given the questionnaire by a support worker, receptionist or sonographer to complete while waiting for their anomaly scan. Once completed, the questionnaires were placed in a box in the ultrasound department. Due to logistic reasons, one Health Board distributed and collected the questionnaires when the women attended their first hospital visit.

**Table 2:** Data items collected for the study and source

**Data item**	**Source**
**Data collected specifically for this study**	
Name, date of birth, address, postcode	Baseline questionnaire, RADIS2
NHS number	
Employment status	Baseline questionnaire
Smoking and alcohol use	
Obstetric history	
Weight	RADIS2 or paper proforma
Height	
BMI	
Presence or absence of the following markers:	
Cardiac Echogenic Foci, Choroid Plexus Cyst, Echogenic Bowel, Mild Ventriculomegaly, Nuchal Thickening, Short Femur, Pelvicalyceal Dilatation	
**Routinely collected data**	
Pregnancy outcome, live birth, stillbirth, gestation at birth, birth weight, neonatal death, infant death	NCCHD and AWPS
Presence of any structural abnormality, congenital malformation or cystic fibrosis	CARIS
Cytogenetic information	Cardiff and Vale Cytogenetic laboratory, CARIS
Down’s syndrome risk	NHS Biochemistry laboratory

#### *Data collection on markers*

The definitions of the seven markers included in the study are shown in Table 1. The current practice in Wales is to routinely report four of these (echogenic bowel, mild to moderate ventriculomegaly, renal pelvicalyceal dilatation, and nuchal thickening). Data on the other three markers (choroid plexus cysts, echogenic cardiac foci, and short femur) were collected specifically for this study; these were not recorded on the clinical report and the woman was not informed of the finding given that these markers would not be reported in usual care. This was explained to the women as part of the consent process.

The presence or absence of markers was recorded in the RadIS2 system at the time of the 18 to 20 week scan. An additional reporting screen was added on to this system to enable rapid and accurate data collection for the study. Sonographers were requested to access this screen during the scans of all women who had consented to participate. RadIS2 implementation was delayed in three Health Boards. Alternative data reporting arrangements using paper forms were implemented at these locations.

#### *Quality assurance of marker reporting*

Before recruitment began, the sonographers in each Health Board (n = 140) attended an introductory session and were provided with a ‘Reference Guide for Sonographers’ which included the criteria for each marker as set out for this study. After this session, they were asked to complete a paper-based questionnaire to assess (i) compliance with the study protocol for the measurement of markers, (ii) agreement between sonographers and (iii) intra-sonographer agreement in reporting markers (by asking a sample of sonographers to complete the questionnaire a second time). The data generated were used to assess the level of compliance to the study protocol for the reporting of markers. These training and educational issues were discussed with superintendent sonographers in each Health Board who then provided additional training for their sonographers to address any gaps in knowledge identified.

An expert panel, consisting of radiologists and superintendent sonographers was established to review all anomaly scan images in which a marker had been reported (where images were available for review), to confirm the presence of the reported marker and identify any false positives. A review of all scan data by the expert panel to assess for false negative reports was not possible due to the substantial number of scans. However, the panel did review the scan images for all stillbirths, chromosomal abnormalities reported (Down’s and Edward’s syndrome) and cases of cystic fibrosis, to ascertain whether or not there were any markers visible in those scans that were not reported initially.

#### *Definitions of adverse pregnancy outcomes*

Major congenital abnormalities were as defined in Chapter XVII of the tenth revision of the International Statistical Classification of Diseases and Related Health Problems [23] and reported to the European Surveillance of Congenital Anomalies (EUROCAT), and were ascertained from cytogenetic results following amniocentesis or a report to CARIS at up to 1 year after the birth. Stillbirths are defined as the *in-utero* death of a baby after 24 completed weeks’ gestation. A preterm birth is defined as a birth before 37 weeks’ gestation, and small for gestational age is defined as below the 3^rd^ centile for birth weight appropriate for gestational age, stratified by sex.

#### *Data linkage*

We used the NHS numbers of study participants to link the ultrasound scan data with routinely-collected data on pregnancy and health outcomes from NCCHD, AWPS, CARIS, All Wales Medical Genetics Service, Cytogenetics Department and the Down’s screening service (University Hospital of Wales, Department of Medical Biochemistry and Immunology). The extraction of outcome data and linkage to scan data using NHS numbers was co-ordinated by the NHS Wales Informatics Service (NWIS) (work completed during 2012 and 2013). All baseline questionnaires and ultrasound data that were collected on the paper forms were entered on a database by Screening Services Wales and uploaded to NWIS to be linked to the other study datasets.

#### *Statistical analysis*

We will calculate study recruitment rates for each hospital and Health Board. Socio-demographic characteristics (age at pregnancy, employment status, cigarette smoking, alcohol use and the Welsh Index of Multiple Deprivation) and pregnancy outcomes of women recruited to the study will be described. Characteristics of women recruited to the study will be compared with data routinely reported for pregnant women in Wales, to assess how representative the sample is of the general population.

We will calculate the reported prevalence of each marker as a proportion of the number of pregnant women with singleton pregnancies with scan data. We will also report the prevalence of validated markers (defined as markers confirmed by the expert panel). 95% confidence intervals [CI] for the validated markers will be estimated using Bayesian methods in Winbugs, to account for the two-stage sampling process (the availability of the images for review and the proportion of markers reviewed that were confirmed). We will describe the variation in prevalence of validated markers by NHS hospital and Health Board as well as by maternal socio-demographic and clinical characteristics. Unadjusted risk ratios with 95% CI will be calculated to estimate the risk of the adverse pregnancy outcomes in the presence of a validated marker compared with no marker, and in the presence of two or more markers compared with no marker. Odds ratios, adjusted for maternal age and deprivation score, will be calculated using logistic regression models if there is evidence of confounding by these variables. Data will be analysed using Stata version 13.0.

## Discussion

This is a large prospective population-based study designed to estimate the prevalence of markers in a cohort of pregnant women and investigate associations with abnormal pregnancy outcomes. Quantifying these markers and assessing their significance is important as it will inform pregnancy management and parental counselling. The study addresses the criticism that many previous studies have not described their populations well and may not have included “all-risk” women [4]. This work has established a cohort of babies for longer-term follow-up to further explore associations between specific markers and child health outcomes, which are currently poorly understood [3].

The study takes advantage of an electronic information system for the collection of radiological data that was recently implemented in parts of Wales. This allows for the linkage of these data to high-quality population-based registers of pregnancy outcomes, congenital anomalies, and stillbirths. A major strength of this study is that we asked pregnant women at the time of recruitment for permission to access data that is routinely collected about the health of their baby to facilitate follow-up studies investigating health outcomes associated with markers detected at the 18-20 week anomaly scan. This provides some flexibility in the design of future follow-up studies, allowing us to utilise the wealth of health data that is routinely collected in a cost-effective way. For example, using record linkage to routinely collected healthcare datasets we could establish a future e-cohort study to explore whether babies with a renal marker detected during pregnancy have a higher risk of urinary tract infections or hospital admissions for renal disease during childhood, compared to babies without a marker. This information is vital for pregnant women, families and clinicians to facilitate the development of appropriate clinical guidelines and care pathways during pregnancy and after birth, including the appropriate use of medical interventions and treatment.

## Abbreviations

AP: Anterior-posterior; AWPS: All Wales Perinatal Survey; BPD: Biparietal diameter; CARIS: Congenital Anomaly Register and Information Service; CRC Cymru: Clinical Research Collaboration Cymru; EUROCAT: European Surveillance of Congenital Anomalies; NCCHD: National Community Child Health Database; NHS: National Health Service; NICE: National Institute for Health and Care Excellence; NWIS: NHS Wales Informatics Service; RadIS2: Radiological Information System (an electronic information system for radiological data storage and reporting in Wales).

## Competing interests

The authors declare that we have no competing interests.

## Authors’ contributions

SP, ST, CD, FD, DF and LC designed the study. CD had the original idea for the study, provided professional advice on the obstetric ultrasound and markers, was involved with the development of Radis2 marker screen for the study and provided oversight for the training of sonographers and the quality assurance process. FD and MW provided statistical expertise, DF provided epidemiological expertise and LC provided professional advice on obstetric ultrasound, fetal medicine and genetics and relevance to clinical practice. MW was responsible for the production of a linked study database. MAW provided advice on the women’s perspective in the study design, implementation and interpretation of data. ST and SP were responsible for the co-ordination and management of the study, analysed the data and produced the quality assurance report. SP and LH wrote the first draft of this paper. All authors contributed to the text of the paper, subsequent revisions and the final version of the paper. All authors read and approved the final manuscript.

## Pre-publication history

The pre-publication history for this paper can be accessed here:

http://www.biomedcentral.com/1471-2393/14/164/prepub

## Supplementary Material

Additional file 1 The consent form. Click here for file

Additional file 2 The patient information leaflet. Click here for file
