# Supplementary material for: The Welsh study of mothers and babies: protocol for a population-based cohort study to investigate the clinical significance of defined ultrasound findings of uncertain significance
Source: BMC Pregnancy Childbirth. 2014 May 8;14:164. doi: 10.1186/1471-2393-14-164 (PMC4029820; doi:10.1186/1471-2393-14-164)
Supplement: Additional file 1 — The consent form. [file 1471-2393-14-164-S1.pdf]

Welsh Study of Mothers and Babies

Yr Astudiaeth o Famau a Babanod yng Nghymru

NHS Number or Addressograph
Rhif GIG neu Addressograph

Funded by the Welsh Assembly Government/Medical Research Council Health Research Award Partnership
Ariannwyd trwy Bartneriaeth Dyfarniadau Ymchwil Iechyd Llywodraeth Cynulliad Cymru/Cyngor Ymchwil Feddygol

Ffurflen ganiatâd

Rhowch gylch o gwmpas **Ydw** neu **Na**

Rydw i'n cadarnhau fy mod i wedi darllen a deall y daflen wybodaeth 'Yr Astudiaeth o Famau a Babanod yng Nghymru' (rhifyn 1: Ebrill 2008).	Ydw	Na
Rydw i wedi cael cyfle i feddwl am y wybodaeth ac i ofyn cwestiynau, ac rydw i'n fodlon gyda'r atebion a gefais i fy nghwestiynau.	Ydw	Na
**Rydw i'n cytuno i gymryd rhan yn yr Astudiaeth o Famau a Babanod yng Nghymru.**	Ydw	Na
Rydw i'n deall nad oes rhaid i mi gymryd rhan. Rydw i'n rhydd i dynnu'n ôl o'r astudiaeth unrhyw bryd, heb orfod rhoi unrhyw reswm, ac ni fydd hyn yn effeithio ar fy ngofal cyn-geni na fy hawliau cyfreithiol.	Ydw	Na
Rydw i'n deall y bydd tîm ymchwil yr astudiaeth yn gweld y wybodaeth a gesglir yn arferol amdanaf i a fy mabi, os yw'n berthnasol i'r ymchwil hwn. Rydw i'n rhoi fy nghaniatâd i'r tîm ymchwil ddefnyddio'r wybodaeth hon gan ddefnyddio fy rhif GIG.	Ydw	Na
Rydw i'n deall y bydd canlyniadau fy mhrawf sgrinio am syndrom Down, os byddaf i'n cael y prawf hwn, ar gael i'r tîm ymchwil.	Ydw	Na
Rydw i'n deall y bydd canlyniadau gweithred amniocentesis, os byddaf i'n cael cynnig un ac yn ei derbyn, ar gael i'r tîm ymchwil.	Ydw	Na
Rydw i'n deall y bydd canlyniadau'r prawf smotyn gwaed y newydd-anedig a fydd yn cael ei wneud ar fy mabi, ar gael i'r tîm ymchwil.	Ydw	Na
Rydw i'n fodlon i'r tîm ymchwil ysgrifennu ataf i ar ôl i fy mabi gael ei eni.	Ydw	Na

Consent form

Please circle **Yes** or **No**

I confirm that I have read and understood the information for women *'Welsh Study of Mothers and Babies'* (edition 1: April 2008).	Yes	No
I have had the chance to think about the information and ask questions, and have had those questions answered satisfactorily.	Yes	No
**I agree to take part in the Welsh Study of Mothers and Babies.**	Yes	No
I understand that I don't have to take part, I am free to withdraw from the study at any time without giving any reason, and that this won't affect my antenatal care or legal rights.	Yes	No
I understand that the information that is routinely collected about me and my baby will be looked at by the study research team, if it is relevant to this research. I give permission for the research team to use this information using my NHS number.	Yes	No
If I accept an antenatal screening test for Down's syndrome, I understand that the results will be available to the research team.	Yes	No
If I am offered and accept an amniocentesis procedure, I understand that the results will be available to the research team.	Yes	No
I understand the results of the newborn blood-spot screening that will be carried out on my baby will be available to the research team.	Yes	No
I agree for the study team to write to me after my baby has been born.	Yes	No

Your Name/Eich Enw   Your Date of Birth/Eich dyddiad geni__ / __ /______

Your Signature/Eich Llofnod   Date/Dyddiad__ / __ /______

Name of person getting consent
Enw'r person sy'n gofyn am ganiatâd

Signature/Llofnod   Date/Dyddiad __ / __ /______

When completed, please give the participant a copy, one copy for the researcher site file, one copy for the sonographer and one copy to be filed in the maternity notes.

Ar ôl i'r ffurflen gael ei llenwi, gwnewch dri chopi ohoni. Rhowch un copi i'r fam, un copi i'r sonograffydd, cadwch un copi mewn ffeil ar safle'r ymchwilydd a ffeiliwch y ffurflen wreiddiol yn y nodiadau mamolaeth.
