# Supplementary material for: The Welsh study of mothers and babies: protocol for a population-based cohort study to investigate the clinical significance of defined ultrasound findings of uncertain significance
Source: BMC Pregnancy Childbirth. 2014 May 8;14:164. doi: 10.1186/1471-2393-14-164 (PMC4029820; doi:10.1186/1471-2393-14-164)
Supplement: Additional file 2 — The patient information leaflet. [file 1471-2393-14-164-S2.pdf]

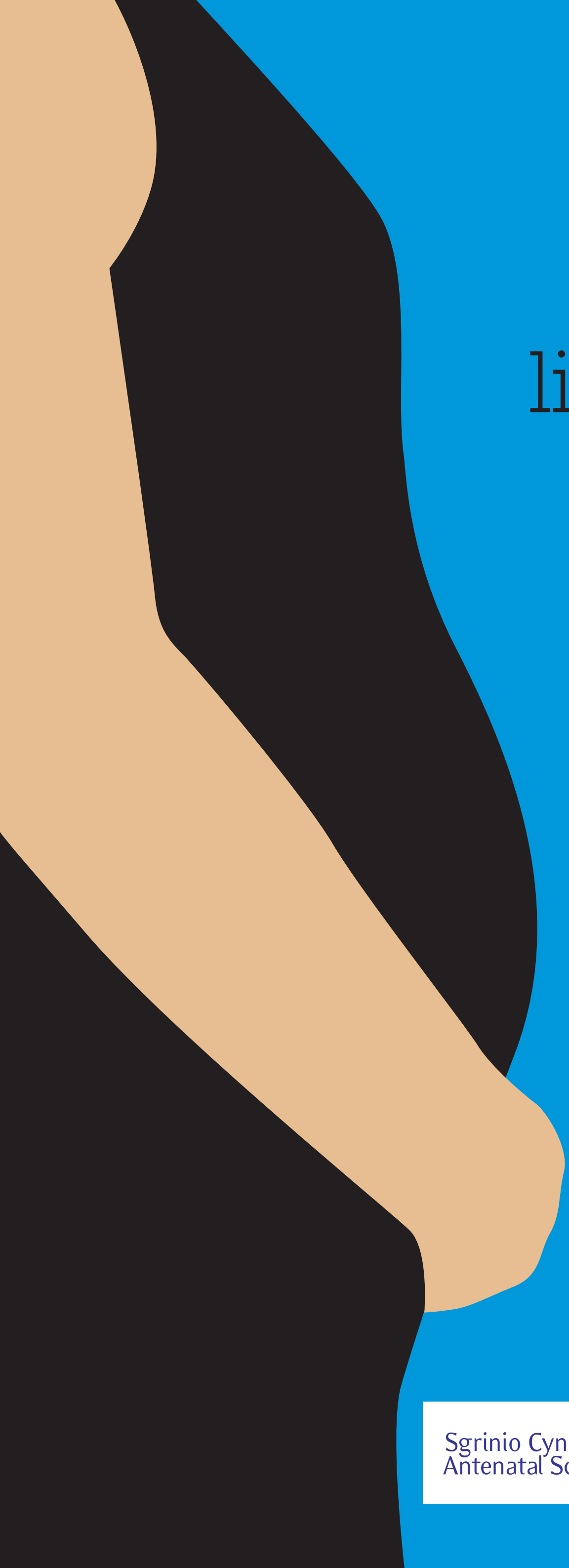

Information for women

We would like to invite you to take part in the **Welsh study of mothers and babies**

Sgrinio Cyn Geni Cymru
Antenatal Screening Wales

CARDIFF UNIVERSITY
PRIFYSGOL CAERDYDD

We would like to invite you to take part in the **Welsh study of mothers and babies.** Before you decide, you need to understand why we are carrying out this research and what it would involve for you. Please take time to read this information sheet carefully.

If you would like more information about the study then ask your midwife. If you decide you do not want to take part, it will not affect the care that you receive during your pregnancy. Please take your time to decide if you would like to take part in the study. You can stop taking part in the study at any time, even if you have agreed to take part.

What is the purpose of the study?

The purpose of this study is to investigate whether certain findings that are sometimes seen on ultrasound scans done between 18 and 20 weeks of pregnancy can tell us anything about the health of your baby. These findings are often known as 'markers'. As ultrasound images have improved, more of the ultrasound markers have been found. However, we do not know if these markers are important for your baby's health. So we need to collect this information and investigate if there are any connections with long-term health and development of the baby.

Why have I been invited?

We are asking all women in Wales who have decided to have an ultrasound scan at 18 to 20 weeks to take part in the study.

Crystal Mark 16313
Clarity approved by Plain English Campaign

www.wsmb.org.uk
April 2008
WSMB 1st edition

Do I have to take part?

It is entirely up to you if you would like to take part in the study. You do not have to take part in the study and you will be able to withdraw at any time without giving a reason. This would not affect the standard of care you receive.

What will happen to me if I take part?

- We will ask you to fill in a short questionnaire.
- If you agree to take part, the person doing the ultrasound scan will record on the computer screen whether or not they have seen any markers. They will send the information to the study centre.
- The person doing the scan will tell you if they have seen any problems that are normally reported at the time.
- They will not tell you about ultrasound markers that are not normally reported because we don't yet know what they mean.
- The research team will use your NHS number to link your scan records with results from other tests you might have during pregnancy.
- The research team will use your baby's NHS number to collect information about the health of your baby and to get the results of your baby's blood-spot test.
- The study team will write to you after the birth to check on the health of your baby.
- If you would like to take part in this study, we will ask you to sign a consent form.

What will I have to do?

Come for your ultrasound scan appointment as arranged with you by the Ultrasound Department. The Antenatal Screening Wales leaflet, *'Ultrasound scans in pregnancy'* gives you more information about the scan.

Will my private and medical details be kept safe?

All information the team collect about you during the study will be kept confidential. All information will be held securely on hospital computers and by the study centre.

What will happen to the results of the research study?

The research team will publish the findings from this study in a scientific journal. They will not include your name and personal details in any reports or publications.

Who is organising and funding the research?

Antenatal Screening Wales is working closely with Cardiff University to carry out this research. It is being funded by the Welsh Assembly Government /Medical Research Council Health Research Award Partnership.

More information and contact details

Your local trust contact is

Name and contact number

Further information also available from the study research midwife

Name and contact number

Lynne Williams
Tel: (029) 2078 7837

A fydd fy manylion personol a meddygol yn cael eu cadw'n saff?

Bydd yr holl wybodaeth amdanoch chi y mae'r tîm yn ei chasglu yn ystod yr astudiaeth yn cael ei chadw'n gyfrinachol. Bydd y wybodaeth yn cael ei chadw'n ddiogel ar gyfrifiaduron ysbytai ac yng nghanolfan yr astudiaeth.

Beth fydd yn digwydd i ganlyniadau'r astudiaeth ymchwil?

Bydd y tîm ymchwilio'n cyhoeddi darganfyddiadau'r astudiaeth hon mewn cylchgrawn gwyddonol. Ni fyddan nhw'n cynnwys eich enw na'ch manylion personol mewn unrhyw adroddiadau na chyhoeddiadau.

Pwy sy'n trefnu ac yn ariannu'r ymchwil?

Mae Sgrinio Cyn Geni Cymru'n gweithio'n agos gyda Phrifysgol Caerdydd i wneud yr ymchwil. Mae'n cael ei ariannu trwy Bartneriaeth Dyfarniadau Ymchwil Iechyd Llywodraeth Cynulliad Cymru/Cyngor Ymchwil Feddygol.

Mwy o wybodaeth a manylion cyswllt

Y cyswllt yn eich ymddiriedolaeth leol yw

Enw a rhif cyswllt

Mae mwy o wybodaeth ar gael hefyd gan fydwraig ymchwil yr astudiaeth

Enw a rhif cyswllt

Lynne Williams
Ffôn: (029) 2078 7837

Oes rhaid i mi gymryd rhan?

Rydych chi’n gwbl rydd i benderfynu ydych chi am gymryd rhan yn yr astudiaeth. Nid oes rhaid i chi gymryd rhan ynddi a byddwch chi’n gwbl rydd i dynnu’n ôl unrhyw bryd, heb orfod rhoi rheswm. Ni fyddai hyn yn effeithio ar safon eich gofal.

Beth fydd yn digwydd os byddaf i’n cymryd rhan?

- Byddwn ni’n gofyn i chi lenwi holiadur byr.
- Os byddwch chi’n cytuno i gymryd rhan, bydd y sawl sy’n gwneud y sgan uwchsain yn cofnodi ar sgrin y cyfrifiadur a yw wedi gweld unrhyw farcwyr ai peidio. Byddan nhw’n anfon y wybodaeth i ganolfan yr astudiaeth.
- Bydd y sawl sy’n gwneud y sgan yn dweud wrthych chi a ydyn nhw wedi gweld unrhyw broblemau sydd fel arfer yn cael eu cofnodi ar y pryd.
- Ni fyddan nhw’n dweud wrthych chi am farcwyr uwchsain sydd ddim fel arfer yn cael eu cofnodi gan ein bod ni’n ansicr ar hyn o bryd beth yw eu harwyddocâd.
- Bydd y tîm ymchwilio’n defnyddio’ch rhif GIG i gysylltu cofnodion eich sganiau â chanlyniadau unrhyw brofion eraill y gallwch chi eu cael yn ystod eich beichiogrwydd.
- Bydd y tîm ymchwilio’n defnyddio rhif GIG eich babi i gasglu gwybodaeth am ei iechyd ac i gael canlyniadau ei brawf smotyn gwaed.
- Bydd y tîm ymchwilio’n ysgrifennu atoch chi ar ôl y geni i wirio iechyd eich babi.
- Os ydych chi’n awyddus i gymryd rhan yn yr astudiaeth hon, byddwn ni’n gofyn i chi lofnodi ffurflen ganiatâd.

Beth fydd rhaid i mi ei wneud?

Dod i gael y sgan uwchsain y mae’r adran uwchsain wedi’i drefnu gyda chi. Mae mwy o wybodaeth am y sgan yn nhaflen Sgrinio Cyn Geni Cymru *‘Sganiau uwchsain mewn beichiogrwydd’*.

Hoffwn eich gwahodd i gymryd rhan yn yr astudiaeth o famau a babanod yng Nghymru. Cyn i chi benderfynu, mae angen i chi ddeall pam rydyn ni'n gwneud yr ymchwil a beth y byddai'n ei olygu i chi. Darllenwch y wybodaeth yn y daflen hon yn ofalus.

Os hoffech chi gael mwy o wybodaeth am yr astudiaeth, gofynnwch i'ch bydwraig. Os byddwch chi'n penderfynu peidio â chymryd rhan, ni fydd hynny'n effeithio ar y gofal y byddwch chi'n ei gael yn ystod eich beichiogrwydd. Cymerwch eich amser i benderfynu a ydych chi am gymryd rhan yn yr astudiaeth neu beidio. Gallwch chi dynnu'n ôl o'r astudiaeth unrhyw bryd, hyd yn oed os ydych chi wedi cytuno i gymryd rhan.

Beth yw pwrpas yr astudiaeth?

Pwrpas yr astudiaeth hon yw ceisio canfod a yw rhai darganfyddiadau sydd i'w gweld ar y sganiau uwchsain sy'n cael eu gwneud rhwng wythnosau 18-20 o feichiogrwydd yn dweud unrhyw beth wrthyn ni am iechyd y babi. Mae'r darganfyddiadau weithiau'n cael eu galw'n 'farcwyr'. Wrth i ddelweddau uwchsain wella, mae mwy o'r marcwyr uwchsain wedi cael eu canfod. Ond nid ydyn ni'n gwybod a yw'r marcwyr hyn yn bwysig i iechyd eich babi. Mae angen i ni, felly, gasglu'r wybodaeth hon a phwyso a mesur a oes unrhyw gysylltiadau ag iechyd a datblygiad y babi yn y tymor hir.

Pam ydych chi wedi fy ngwahodd i?

Rydyn ni'n gofyn i bob menyw yng Nghymru sydd wedi penderfynu cael sgan uwchsain yn ystod wythnosau 18-20 gymryd rhan yn yr astudiaeth.

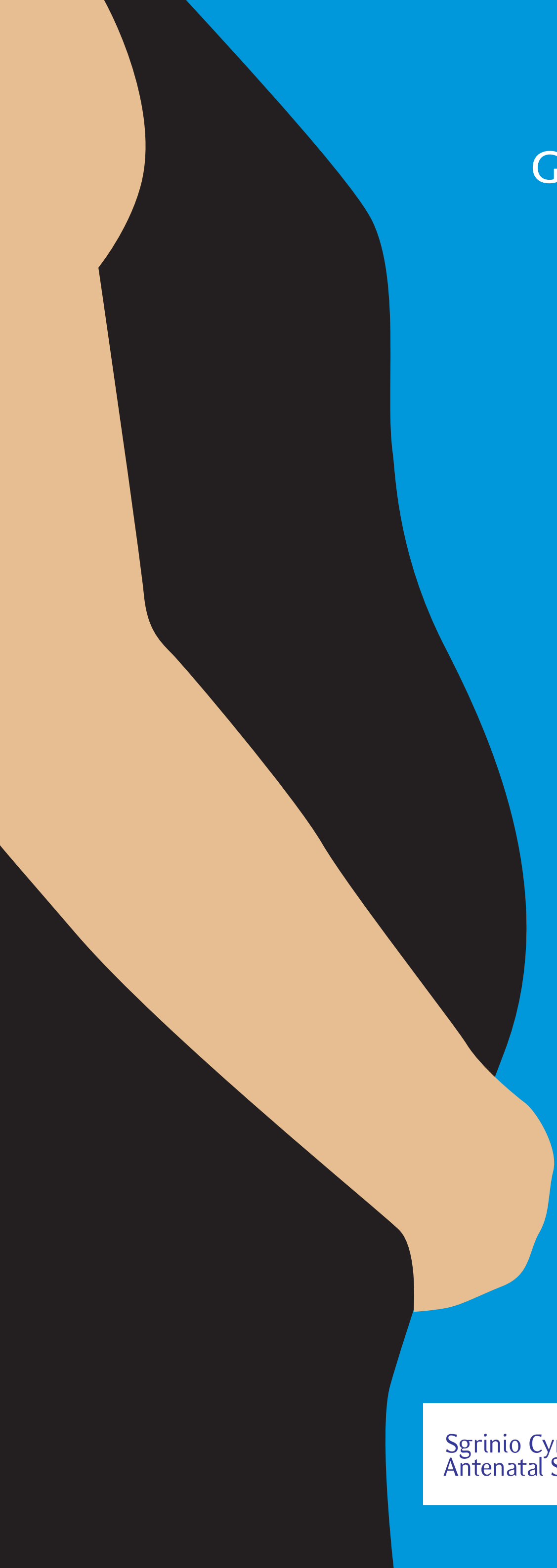

Gwybodaeth i fenywod

Hoffwn eich gwahodd i gymryd rhan yn yr **astudiaeth o famau a babanod yng Nghymru**

Sgrinio Cyn Geni Cymru
Antenatal Screening Wales

Cardiff University
Prifysgol Caerdydd
